# Supplementary material for: Linking Affect Dynamics and Well-Being: A Novel Methodological Approach for Mental Health
Source: Healthcare (Basel). 2024 Aug 24;12(17):1690. doi: 10.3390/healthcare12171690 (PMC11395663; doi:10.3390/healthcare12171690)
Supplement: Supplementary file 1 [file healthcare-12-01690-s001.zip › healthcare-3160587-supplementary.pdf]

Supplementary Materials

Table S1. Correlation matrix. Facial EMG1/EMG2 ratio's transitions, CI confidence interval (95% CI are indicated as the inferior and superior values of the interval), ρ Spearman's correlation coefficient, p value.

	Pair	Correlation	P_Value	Lower_CI	Upper_CI
BDI	AB_EMG1/EMG2	0.3173889	0.04054694	0.008891055	0.5644982
	BA_EMG1/EMG2	0.2669543	0.08744709	-0.100563297	0.5716927
	CD_EMG1/EMG2	0.3143840	0.04259198	0.010365793	0.5796806
	DC_EMG1/EMG2	0.2788117	0.07377273	-0.049151416	0.5309828
	AC_EMG1/EMG2	0.1152443	0.4673754	-0.211160641	0.4390742
	CA_EMG1/EMG2	0.1746939	0.268504	-0.128810727	0.4535097
	BD_EMG1/EMG2	0.2487621	0.1121515	-0.113718858	0.5401452
	DB_EMG1/EMG2	0.3644125	0.01766248	0.061933047	0.6009857
	AD_EMG1/EMG2	0.0459678	0.7725295	-0.253340436	0.3136244
	DA_EMG1/EMG2	0.3202315	0.03868683	-0.012169390	0.5833569
	BC_EMG1/EMG2	0.3190945	0.03942233	0.016255174	0.5759305
	CB_EMG1/EMG2	0.3825235	0.01241517	0.084246449	0.6238492

	Pair	Correlation	P_Value	Lower_CI	Upper_CI
CDS	AB_EMG1/EMG2	0.3179866	0.04014994	0.030268706	0.5514050
	BA_EMG1/EMG2	0.1626814	0.3033125	-0.143137583	0.4558530
	CD_EMG1/EMG2	0.2344168	0.1351086	-0.061055650	0.5090948
	DC_EMG1/EMG2	0.2369296	0.1308537	-0.059440024	0.5210760
	AC_EMG1/EMG2	0.1471995	0.3522398	-0.168041587	0.4627219
	CA_EMG1/EMG2	0.2453595	0.1173084	-0.058843319	0.5210979
	BD_EMG1/EMG2	0.2649753	0.08990941	-0.053041996	0.5611917
	DB_EMG1/EMG2	0.3141769	0.04273596	0.003457894	0.5723056
	AD_EMG1/EMG2	0.1350409	0.3938426	-0.189123388	0.4617902
	DA_EMG1/EMG2	0.1677069	0.2884144	-0.159580824	0.4784684
	BC_EMG1/EMG2	0.1373105	0.3858672	-0.154515146	0.4076407
	CB_EMG1/EMG2	0.3177434	0.0403111	0.048636907	0.5624552

	Pair	Correlation	P_Value	Lower_CI	Upper_CI
Mind Reading (CDS)	AB_EMG1/EMG2	0.0008223909	0.9958759	-0.30203584	0.2902017
	BA_EMG1/EMG2	0.0067436053	0.9661917	-0.28366151	0.3259861
	CD_EMG1/EMG2	-0.0385701326	0.8083853	-0.34672955	0.3197013
	DC_EMG1/EMG2	-0.0204775331	0.8975808	-0.33899618	0.3166523
	AC_EMG1/EMG2	-0.0692453128	0.6630242	-0.39879197	0.2588688
	CA_EMG1/EMG2	0.0673538137	0.6717034	-0.27478811	0.3972259
	BD_EMG1/EMG2	0.1481948380	0.3489571	-0.17882965	0.4622923
	DB_EMG1/EMG2	0.1521423143	0.336123	-0.16136306	0.4462741
	AD_EMG1/EMG2	-0.0152142314	0.923815	-0.36260994	0.3046692
	DA_EMG1/EMG2	-0.0151319923	0.9242256	-0.34125456	0.3261432
	BC_EMG1/EMG2	-0.0986869066	0.534084	-0.39871930	0.2251321

	CB_EMG1/EMG2	0.0802653507	0.6133501	-0.24093167	0.3942772

Pair		Correlation	P_Value	Lower_CI	Upper_CI
Catastrophizing (CDS)	AB_EMG1/EMG2	0.3494356	0.02331146	0.03218293	0.5937959
	BA_EMG1/EMG2	0.2214286	0.1587531	-0.09830524	0.4965011
	CD_EMG1/EMG2	0.2677324	0.08649341	-0.04274346	0.5381559
	DC_EMG1/EMG2	0.2948312	0.05803485	-0.03564478	0.5755037
	AC_EMG1/EMG2	0.1923767	0.2222531	-0.13209141	0.4988579
	CA_EMG1/EMG2	0.2640704	0.09105296	-0.04725191	0.5346233
	BD_EMG1/EMG2	0.3834514	0.01218666	0.04989199	0.6559820
	DB_EMG1/EMG2	0.2688717	0.08511167	-0.02383107	0.5361031
	AD_EMG1/EMG2	0.1340290	0.3974293	-0.18596178	0.4159832
	DA_EMG1/EMG2	0.3325090	0.03143606	-0.05817149	0.6168323
	BC_EMG1/EMG2	0.2310311	0.141003	-0.07063657	0.5133539
	CB_EMG1/EMG2	0.3678269	0.01655066	0.06329183	0.6096989

Pair		Correlation	P_Value	Lower_CI	Upper_CI
All or Nothing Thinking (CDS)	AB_EMG1/EMG2	0.2639411	0.09121732	-0.08568676	0.5717771
	BA_EMG1/EMG2	0.2815645	0.07085261	-0.01370241	0.5465708
	CD_EMG1/EMG2	0.1931196	0.2204385	-0.15858155	0.4751728
	DC_EMG1/EMG2	0.2532851	0.1055652	-0.09307737	0.5409385
	AC_EMG1/EMG2	0.1396757	0.3776579	-0.17789184	0.4450042
	CA_EMG1/EMG2	0.1352493	0.3931063	-0.22780998	0.4617774
	BD_EMG1/EMG2	0.3467301	0.02447772	0.05017043	0.6084318
	DB_EMG1/EMG2	0.1871359	0.2353469	-0.14483165	0.4648313
	AD_EMG1/EMG2	0.1125438	0.4779426	-0.22273448	0.4095384
	DA_EMG1/EMG2	0.2120546	0.177598	-0.10276898	0.4998966
	BC_EMG1/EMG2	0.1811521	0.2509273	-0.13932524	0.4500586
	CB_EMG1/EMG2	0.1551679	0.3264869	-0.17386660	0.4556636

Pair		Correlation	P_Value	Lower_CI	Upper_CI
Emotional Reasoning (CDS)	AB_EMG1/EMG2	0.14248279	0.3680501	-0.13485109	0.4397871
	BA_EMG1/EMG2	0.16670897	0.2913343	-0.14240505	0.4696639
	CD_EMG1/EMG2	0.20267869	0.1979978	-0.08064494	0.4976853
	DC_EMG1/EMG2	0.19348096	0.2195597	-0.09834220	0.4951836
	AC_EMG1/EMG2	-0.02299434	0.8850724	-0.33633390	0.3094856
	CA_EMG1/EMG2	0.22296299	0.1558126	-0.09875083	0.5446966
	BD_EMG1/EMG2	0.22862945	0.1452981	-0.07290114	0.5412192
	DB_EMG1/EMG2	0.24940648	0.1111946	-0.06417570	0.5491239
	AD_EMG1/EMG2	0.05329760	0.7374573	-0.25354800	0.3757623
	DA_EMG1/EMG2	0.15660789	0.3219618	-0.15858335	0.4657667
	BC_EMG1/EMG2	0.08540755	0.5907258	-0.23635869	0.4067901
	CB_EMG1/EMG2	0.20358204	0.1959635	-0.09975040	0.4943783

Pair		Correlation	P_Value	Lower_CI	Upper_CI

Labeling (CDS)	AB_EMG1/EMG2	0.25760638	0.09955237	-0.013965139	0.4981023
	BA_EMG1/EMG2	0.02160307	0.8919838	-0.286258136	0.3349012
	CD_EMG1/EMG2	0.18382987	0.2438716	-0.114406721	0.4560943
	DC_EMG1/EMG2	0.12570539	0.4276423	-0.169787011	0.4173091
	AC_EMG1/EMG2	0.17730819	0.2612934	-0.139842413	0.4768399
	CA_EMG1/EMG2	0.20910138	0.1838539	-0.104732565	0.4829818
	BD_EMG1/EMG2	0.10516210	0.5074572	-0.214939485	0.4190425
	DB_EMG1/EMG2	0.18293314	0.2462193	-0.115407800	0.4531975
	AD_EMG1/EMG2	0.22075889	0.160049	-0.099596593	0.5138693
	DA_EMG1/EMG2	0.10475449	0.5091133	-0.230059814	0.4049675
	BC_EMG1/EMG2	0.09114049	0.5659478	-0.196373062	0.3726199
	CB_EMG1/EMG2	0.28173660	0.07067312	-0.001098069	0.5398370

Pair		Correlation	P_Value	Lower_CI	Upper_CI
Mental Filter (CDS)	AB_EMG1/EMG2	0.17928675	0.2559227	-0.12804010	0.4814891
	BA_EMG1/EMG2	0.02291022	0.88549	-0.29677212	0.3410639
	CD_EMG1/EMG2	0.04630963	0.7708833	-0.26164434	0.3482603
	DC_EMG1/EMG2	0.02397013	0.88023	-0.27882215	0.3326650
	AC_EMG1/EMG2	0.10444126	0.5103878	-0.23514054	0.4478806
	CA_EMG1/EMG2	0.02201338	0.8899446	-0.26178585	0.3022186
	BD_EMG1/EMG2	0.16974762	0.2825027	-0.15295312	0.4713189
	DB_EMG1/EMG2	0.12311187	0.4373124	-0.19059453	0.4418511
	AD_EMG1/EMG2	-0.08096032	0.610271	-0.39692950	0.2483744
	DA_EMG1/EMG2	0.16029002	0.3105712	-0.16651427	0.4678979
	BC_EMG1/EMG2	-0.00709320	0.9644402	-0.31525428	0.2979149
	CB_EMG1/EMG2	0.21173611	0.1782652	-0.10503166	0.5096186

Pair		Correlation	P_Value	Lower_CI	Upper_CI
Hypergeneralising (CDS)	AB_EMG1/EMG2	0.24923256	0.1114523	-0.04778649	0.5088762
	BA_EMG1/EMG2	0.03399369	0.8307674	-0.27165717	0.3204000
	CD_EMG1/EMG2	0.10770115	0.4972025	-0.17543708	0.4179641
	DC_EMG1/EMG2	0.11783388	0.4573607	-0.19255797	0.4298475
	AC_EMG1/EMG2	0.07027541	0.6583157	-0.27082139	0.4109755
	CA_EMG1/EMG2	0.12551515	0.4283475	-0.18027047	0.4352736
	BD_EMG1/EMG2	0.17724112	0.2614767	-0.14780741	0.4796811
	DB_EMG1/EMG2	0.16784383	0.2880153	-0.12336150	0.4397460
	AD_EMG1/EMG2	0.04976480	0.7543006	-0.24420135	0.3608930
	DA_EMG1/EMG2	0.13491244	0.394297	-0.18019262	0.4360435
	BC_EMG1/EMG2	0.04878421	0.7589961	-0.22363041	0.3332952
	CB_EMG1/EMG2	0.21197025	0.1777745	-0.08332308	0.4849628

Pair		Correlation	P_Value	Lower_CI	Upper_CI
Personalizing (CDS)	AB_EMG1/EMG2	0.078376583	0.6217515	-0.18240093	0.3539703
	BA_EMG1/EMG2	-0.075601447	0.6341818	-0.33709217	0.2195574

	CD_EMG1/EMG2	0.130179132	0.4112466	-0.12668860	0.3943239
	DC_EMG1/EMG2	0.009418645	0.9527936	-0.23481433	0.2816456
	AC_EMG1/EMG2	-0.044149899	0.7813006	-0.33453124	0.2293444
	CA_EMG1/EMG2	0.128833611	0.4161396	-0.18716581	0.4318065
	BD_EMG1/EMG2	-0.169619709	0.2828709	-0.45879396	0.1632290
	DB_EMG1/EMG2	0.145989001	0.3562574	-0.12358444	0.3984966
	AD_EMG1/EMG2	0.182990821	0.2460679	-0.13165221	0.4588829
	DA_EMG1/EMG2	-0.146577666	0.3543002	-0.44555856	0.1878473
	BC_EMG1/EMG2	-0.014296158	0.9283999	-0.29964724	0.2823314
	CB_EMG1/EMG2	0.067696512	0.6701277	-0.20035077	0.3545159
Pair		Correlation	P_Value	Lower_CI	Upper_CI
Should Statements (CDS)	AB_EMG1/EMG2	0.2909262	0.06159842	-0.005217050	0.5382295
	BA_EMG1/EMG2	0.2536301	0.1050753	-0.071223873	0.5302439
	CD_EMG1/EMG2	0.3161174	0.04140227	-0.004110733	0.5764211
	DC_EMG1/EMG2	0.3323118	0.03154297	0.034884311	0.6006131
	AC_EMG1/EMG2	0.2317104	0.1398054	-0.058827722	0.5130418
	CA_EMG1/EMG2	0.3073659	0.04769592	-0.003100581	0.5797702
	BD_EMG1/EMG2	0.2716238	0.08184471	-0.049237263	0.5612298
	DB_EMG1/EMG2	0.3561944	0.0205991	0.033575298	0.6004153
	AD_EMG1/EMG2	0.2343277	0.1352614	-0.073285645	0.5158316
	DA_EMG1/EMG2	0.1970315	0.2110519	-0.137508222	0.4846186
	BC_EMG1/EMG2	0.2041473	0.1946982	-0.097554040	0.4620592
	CB_EMG1/EMG2	0.3579120	0.01995367	0.079180326	0.6065648
Pair		Correlation	P_Value	Lower_CI	Upper_CI
Minimizing (CDS)	AB_EMG1/EMG2	0.3017653	0.05211448	-0.004552302	0.5531068
	BA_EMG1/EMG2	0.2906100	0.0618944	-0.052808625	0.5650694
	CD_EMG1/EMG2	0.3644314	0.01765618	0.066888633	0.6275967
	DC_EMG1/EMG2	0.3216150	0.03780704	0.016554152	0.5738772
	AC_EMG1/EMG2	0.0273139	0.8636701	-0.277014203	0.3305345
	CA_EMG1/EMG2	0.1886546	0.2314998	-0.133862787	0.4819976
	BD_EMG1/EMG2	0.2977461	0.05548376	-0.022888226	0.5717973
	DB_EMG1/EMG2	0.4070017	0.007473459	0.131706644	0.6462463
	AD_EMG1/EMG2	0.1610946	0.3081169	-0.167208265	0.4649062
	DA_EMG1/EMG2	0.3858396	0.01161498	0.109704038	0.6140722
	BC_EMG1/EMG2	0.3116081	0.04455491	0.052113976	0.5527203
	CB_EMG1/EMG2	0.3789496	0.01332969	0.130853710	0.5973107

Table S2. Correlation matrix. Facial EMG1/EMG2 ratios during state-trait, CI confidence interval (95% CI are indicated as the inferior and superior values of the interval), ρ Spearman's correlation coefficient, p value.

	Pair	Correlation	P_Value	Lower_CI	Upper_CI
BDI	AA_EMG1/EMG2	0.1102090	0.4871792	-0.214166191	0.3940382
	BB_EMG1/EMG2	0.3120287	0.04425282	-0.003650332	0.5565034
	CC_EMG1/EMG2	0.2387726	0.127797	-0.101516799	0.5282920
	DD_EMG1/EMG2	0.2743449	0.07871159	-0.045917293	0.5427875
	Pair	Correlation	P_Value	Lower_CI	Upper_CI
CDS	AA_EMG1/EMG2	0.09678204	0.5420441	-0.20238500	0.4073502
	BB_EMG1/EMG2	0.25994976	0.0964035	-0.05415403	0.5399231
	CC_EMG1/EMG2	0.25443788	0.1039347	-0.05539703	0.5120662
	DD_EMG1/EMG2	0.37278108	0.01504292	0.07506821	0.6364089
	Pair	Correlation	P_Value	Lower_CI	Upper_CI
Mind Reading (CDS)	AA_EMG1/EMG2	-0.11283203	0.4768089	-0.41360938	0.2077925
	BB_EMG1/EMG2	0.13141806	0.4067703	-0.19782880	0.4547681
	CC_EMG1/EMG2	0.05649825	0.7223014	-0.27235221	0.3678402
	DD_EMG1/EMG2	0.26045119	0.09573977	-0.03938487	0.5388676
	Pair	Correlation	P_Value	Lower_CI	Upper_CI
Catastrophizing (CDS)	AA_EMG1/EMG2	0.1451777	0.3589655	-0.17043800	0.4487370
	BB_EMG1/EMG2	0.3721399	0.01523124	0.07410548	0.6216374
	CC_EMG1/EMG2	0.2805901	0.07187564	-0.05199761	0.5419036
	DD_EMG1/EMG2	0.3518769	0.02229924	0.05588428	0.5994657
	Pair	Correlation	P_Value	Lower_CI	Upper_CI
All or Nothing Thinking (CDS)	AA_EMG1/EMG2	0.2376290	0.1296874	-0.10225566	0.5147446
	BB_EMG1/EMG2	0.2732856	0.07991993	-0.05748531	0.5458152
	CC_EMG1/EMG2	0.1947590	0.2164704	-0.13474298	0.4793832
	DD_EMG1/EMG2	0.4442735	0.003203687	0.17345290	0.6423682
	Pair	Correlation	P_Value	Lower_CI	Upper_CI
Emotional Reasoning (CDS)	AA_EMG1/EMG2	0.08466845	0.5939549	-0.218978900	0.4121844
	BB_EMG1/EMG2	0.27199021	0.08141726	-0.037556358	0.5526522
	CC_EMG1/EMG2	0.27182596	0.08160866	-0.007529628	0.5522365
	DD_EMG1/EMG2	0.20571723	0.1912139	-0.087874603	0.5072737
	Pair	Correlation	P_Value	Lower_CI	Upper_CI
Labeling (CDS)	AA_EMG1/EMG2	0.0535593	0.7362143	-0.2343920	0.3378254
	BB_EMG1/EMG2	0.1249717	0.4303657	-0.1996419	0.4351283
	CC_EMG1/EMG2	0.1837484	0.2440844	-0.1342270	0.4793092
	DD_EMG1/EMG2	0.2241012	0.1536572	-0.1084361	0.5341397
	Pair	Correlation	P_Value	Lower_CI	Upper_CI
Mental Filter (CDS)	AA_EMG1/EMG2	0.007256262	0.9636232	-0.2939710	0.3169771
	BB_EMG1/EMG2	0.121155122	0.4446877	-0.1724982	0.4170111
	CC_EMG1/EMG2	0.073785589	0.6423696	-0.2366854	0.3701798
	DD_EMG1/EMG2	0.192168656	0.2227632	-0.1136864	0.4615205

Pair		Correlation	P_Value	Lower_CI	Upper_CI
Hypergeneralizing (CDS)	AA_EMG1/EMG2	0.08073501	0.6112685	-0.22829794	0.3823793
	BB_EMG1/EMG2	0.08947857	0.5730811	-0.19968975	0.3928339
	CC_EMG1/EMG2	0.09503523	0.5493934	-0.24027078	0.4254589
	DD_EMG1/EMG2	0.26982489	0.0839689	-0.04029703	0.5459417
Pair		Correlation	P_Value	Lower_CI	Upper_CI
Personalizing (CDS)	AA_EMG1/EMG2	-0.09090675	0.5669486	-0.3447004	0.1821136
	BB_EMG1/EMG2	-0.04078610	0.7976004	-0.3160433	0.2576468
	CC_EMG1/EMG2	0.01782815	0.9107746	-0.2512588	0.3137543
	DD_EMG1/EMG2	-0.08695428	0.5839937	-0.4173538	0.2422520
Pair		Correlation	P_Value	Lower_CI	Upper_CI
Should Statements (CDS)	AA_EMG1/EMG2	0.1266923	0.4239941	-0.17267504	0.4069125
	BB_EMG1/EMG2	0.2958336	0.0571472	-0.03471815	0.5767765
	CC_EMG1/EMG2	0.2846284	0.06771048	-0.03155543	0.5659928
	DD_EMG1/EMG2	0.4258793	0.004923528	0.10911178	0.6668364
Pair		Correlation	P_Value	Lower_CI	Upper_CI
Minimizing (CDS)	AA_EMG1/EMG2	0.1647856	0.2970156	-0.15912073	0.4594846
	BB_EMG1/EMG2	0.3829687	0.01230508	0.10017509	0.6160140
	CC_EMG1/EMG2	0.2879853	0.06439553	-0.01235606	0.5311621
	DD_EMG1/EMG2	0.3909250	0.01047343	0.11944918	0.6045813
